# Code-free machine learning for classification of central nervous system histopathology images

**DOI:** 10.1093/jnen/nlac131

**Published:** 2023-02-03

**Authors:** Patric Jungo, Ekkehard Hewer

**Affiliations:** Institute of Pathology, University of Bern, Bern, Switzerland; Institute of Pathology, University of Bern, Bern, Switzerland

**Keywords:** Astrocytoma, Digital pathology, Glioma, Machine learning, Oligodendroglioma

## Abstract

Machine learning (ML), an application of artificial intelligence, is currently transforming the analysis of biomedical data and specifically of biomedical images including histopathology. The promises of this technology contrast, however, with its currently limited application in routine clinical practice. This discrepancy is in part due to the extent of informatics expertise typically required for implementation of ML. Therefore, we assessed the suitability of 2 publicly accessible code-free ML platforms (Microsoft Custom Vision and Google AutoML), for classification of histopathological images of diagnostic central nervous system tissue samples. When trained with typically 100 to more than 1000 images, both systems were able to perform nontrivial classifications (glioma vs brain metastasis; astrocytoma vs astrocytosis, prediction of 1p/19q co-deletion in IDH-mutant tumors) based on hematoxylin and eosin-stained images with high accuracy (from ∼80% to nearly 100%). External validation of the predicted accuracy and negative control experiments were found to be crucial for verification of the accuracy predicted by the algorithms. Furthermore, we propose a possible diagnostic workflow for pathologists to implement classification of histopathological images based on code-free machine platforms.

## INTRODUCTION

Machine learning (ML) is a paradigm within the broader concept of artificial intelligence characterized by the concept that computer systems develop and refine algorithms themselves based on provided data rather than being explicitly programmed (1). Emerging and predicted future applications of ML in medicine include interpretation of a broad spectrum of clinical and laboratory data as well as radiological and histopathological images. As ML thrives on large, well-annotated datasets, histopathology is regarded as one of its particularly promising targets.

Recently, several ML-based histology applications for screening purposes have received formal approval by health care authorities and are commercially available for prostate cancer (2), and cervical cytology (3). An even broader range of possible applications of ML in histopathology has been suggested in the literature without making the transition to routine diagnostic applications (4–9). Of note, ML approaches are currently also used in pathology for classification of complex nonmorphological data such as methylome data (10).

Nevertheless, there is a marked discrepancy between the promises of ML and its very limited routine application in pathology. Automated screening of gynecological cytology specimens, the currently most widely used application of automated image analysis and arguably one of very few with proven clinical benefit, has previously been based on decades-old algorithms, rather than ML. This lag in routine application of ML in pathology may in part relate to coding competencies required to implement and modify existing ML solutions for utilization in the context of histopathology. The “black box” nature of ML algorithms likely contributes to hesitancy regarding its diagnostic application because it is often impossible for end users to understand why ML algorithms render obviously incorrect results in certain situations.

**Figure 1.**
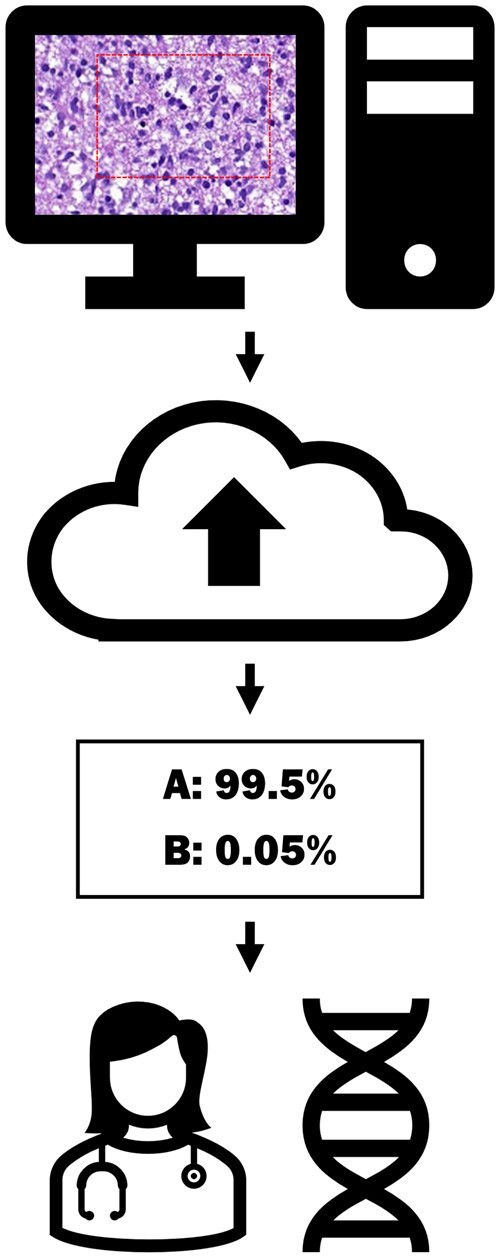
Illustration of a possible diagnostic workflow based on the code-free machine platforms. A region of interest is selected and the image is uploaded. The model returns a prediction. The results are interpreted in the context of the other pertinent data.

This prompted us to assess the suitability of 2 publicly available code-free platforms for ML-based image classification (Microsoft Custom Vision and Google AutoML) for histopathological images. We envisioned a simple workflow (Fig. 1) within which pathologists would define regions of interest (i.e. no image segmentation would be required), and subsequently submit snapshots to the classifier. We reasoned that such an approach might have fundamental benefits as it would: (1) allow end users without substantial coding knowledge to gain hands-on experience with a potent ML tool including the possibility to perform quality and plausibility controls, and (2) might be applied in real-life situations that do not result in regulatory issues because the predictions made by the ML algorithm could be independently verified by other means.

## MATERIALS AND METHODS

### Case selection and reference diagnoses

Cases were identified through full text search of our department’s diagnostic database or included from previous studies (11, 12). Original reports were reviewed and available results of ancillary testing as well as clinical follow-up were included in order to establish respective reference diagnoses as gold standard for image classification. For the purpose of the manuscript, the 2021 WHO Classification of Tumors of the Central Nervous System nomenclature was applied (13). Molecular and immunohistochemical testing had been performed as previously reported (11). Depending on the respective experiments, cases were attributed to 1 of 2 groups (Table 1).

**Table 1.** Definition of groups for each experiment

Experiment	Group 1	Group 2
1. Astrocytoma vs astrocytosis	Group 1 from experiment 3 (79 cases, 770 image files)	Nontumoral, reactive brain tissue (47 cases, 286 image files)
2. Glioma vs brain metastasis	Group 1 and 2 from experiment 3 (121 cases, 1216 image files)	Brain metastases (57 cases, 577 image files)
3. Prediction of 1p/19q-status in IDH-mutant tumors	IDH-mutant astrocytomas (grades II-III) and IDH-mutant glioblastomas (79 cases, 770 image files)	IDH-mutant, 1p/19q co-deleted oligodendrogliomas (grades II-III) (42 cases, 446 image files)
4. Negative control experiment: even vs odd accession numbers	Even accession number (26 cases, 251 image files)	Odd accession number (25 cases, 249 image files)
5. Negative control experiment: astrocytoma vs astrocytoma	Random selection of half of the images of cases from group 1 in experiment 3 (67 cases, 335 images)	Other half of images from the same cases as in group 1 (67 cases, 335 images)

### Generation of histopathological images

**Figure 2.**
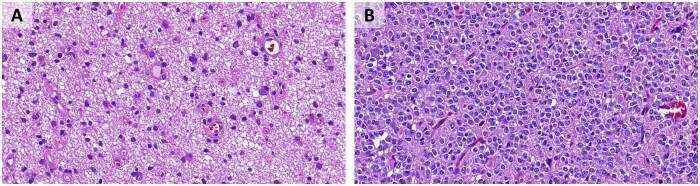
Examples of the image format used for training. **(A)** IDH-mutant astrocytoma. **(B)** IDH-mutant oligodendroglioma, 1p/19q-codeleted.

Hematoxylin and eosin (H&E)-stained slides were scanned with a Pannoramic 250 slide scanner (3DHistech, Budapest, Hungary) with standard settings. Scans (MRXS file format) were reviewed with the CaseViewer (3DHistech) software. Snapshots of representative pathological regions of interest (ROI) of the scan were made at a nominal 400× magnification (40× “objective”) in JPEG file format and measuring 1.920 × 1.017 pixels (Supplementary Data  Fig. S1). Up to 14 representative ROIs reflecting intratumoral heterogeneity were chosen for each case. Examples of 2 such images are given in Figure 2. Slides were reviewed and snapshots were taken without access to diagnostic or other clinical information of the respective cases. The study was performed with approval of the Ethics committee of the Canton of Bern (KEK 2014-200 and KEK 2017-1189).

### Code-free ML

User accounts for Microsoft Custom Vision (14) and Google AutoML (15) were created. Sets of histopathological images were uploaded with the respective user interfaces. Training and analysis were performed with a single tag for each image (single class rather than multi-class mode) even though the tag for a specific image could vary between experiments. For example, the same image might have been labeled as “oligodendroglioma” in the context of prediction of 1p/19q status in IDH-mutant gliomas or “glioma” in the distinction between glioma and gliosis. In order to create our own validation datasets (in addition to the validation datasets defined by the image analysis software), sets of images were excluded from training and subsequently submitted to the respective classifiers.

## RESULTS

### User interfaces

Both platforms offered a relatively straightforward and intuitive user experience. Single images files as well as batches of files could be uploaded and subsequently tagged. Microsoft Custom Vision, in addition to a general model, offered specific models termed “food,” “landmarks,” and “retail.” Preliminary tests showed that all of these performed worse than the general model for classification of histopathological images so these models were not further studied. Even though both platforms underwent several updates over time, the essential functionalities used in our experiments were stable and continued to be available after our first exploratory experiments in Fall 2018. Registration and an initial test phase, which allowed us to perform most of our experiments, were free of charge for both platforms.

After training with a particular set of images, both platforms provide the user with a number of quality parameters for the derived model. These are based on whether or not images from the training set would have been correctly classified with the model that has been obtained throughout multiple iterations. These parameters include overall accuracy (percentage of images which would have been correctly classified across all classes) as well as recall and precision for each class (i.e. percentage of images that would have been correctly attributed to that class; and 1 minus the percentage of images erroneously attributed, respectively). Furthermore, misclassified images can be reviewed. Google AutoML additionally creates an internal validation dataset with each training session for which predictions can be reviewed. Interestingly, it proved difficult to trace back misclassified images as both platforms would only display the image itself, but not the name or other data of the submitted image file. With standard settings and for single-tag classifications, both platforms would choose as optimal compromise between recall and precision a confidence level of 0.5, that is, recall and precision would have the same numerical value.

After training, image files can be submitted to the algorithm. Upon submission of an image file, a classification and quantification of accuracy of fitting with the respective class are returned.

Apart from uploading and tagging images, no particular specifications needed to be made by the user. In particular, the entire interface required no coding for either of the platforms. For each image submitted after the training period, the platforms not only provided a classification, but also a quantitative estimate of the classification’s certainty (ranging from 0% to 100%).

### Training

We uploaded a variety of image datasets of different sizes and compositions. The recommended minimum number of images per class is 100 with Google AutoML, whereas Microsoft Custom Vision does not specify a minimum number of images. Small numbers of training images would result in increased misclassification and overestimated accuracy of classification. We found it important to include heterogeneous examples of each entity and to be aware of what the algorithm had been trained for. Training with few samples of non-neoplastic lung and liver, for example, resulted in an apparently accurate distinction. However, at this stage, an image composed of a small amount of liver tissue on an empty background would be erroneously classified as lung. This indicated that the distinction between lung and liver might have simply been based on the proportion of area covered by tissue. Interestingly, inclusion of only a few images containing small areas of liver with adjacent empty space was sufficient to adequately train the algorithm so that it would not further misinterpret these types of images.

We recurrently observed across multiple experiments that the quality parameters would decrease after inclusion of additional image files. This was particularly true in the range of the minimum numbers of recommended training images and indicated that estimates based on limited numbers of images may have been too optimistic. On the other hand, across the various experiments, we observed a ceiling effect, in that once a certain size of training sets had been reached (typically around 100 to several hundred images), inclusion of additional images would not result in an increased accuracy of predictions.

### Astrocytoma versus astrocytosis

**Figure 3.**
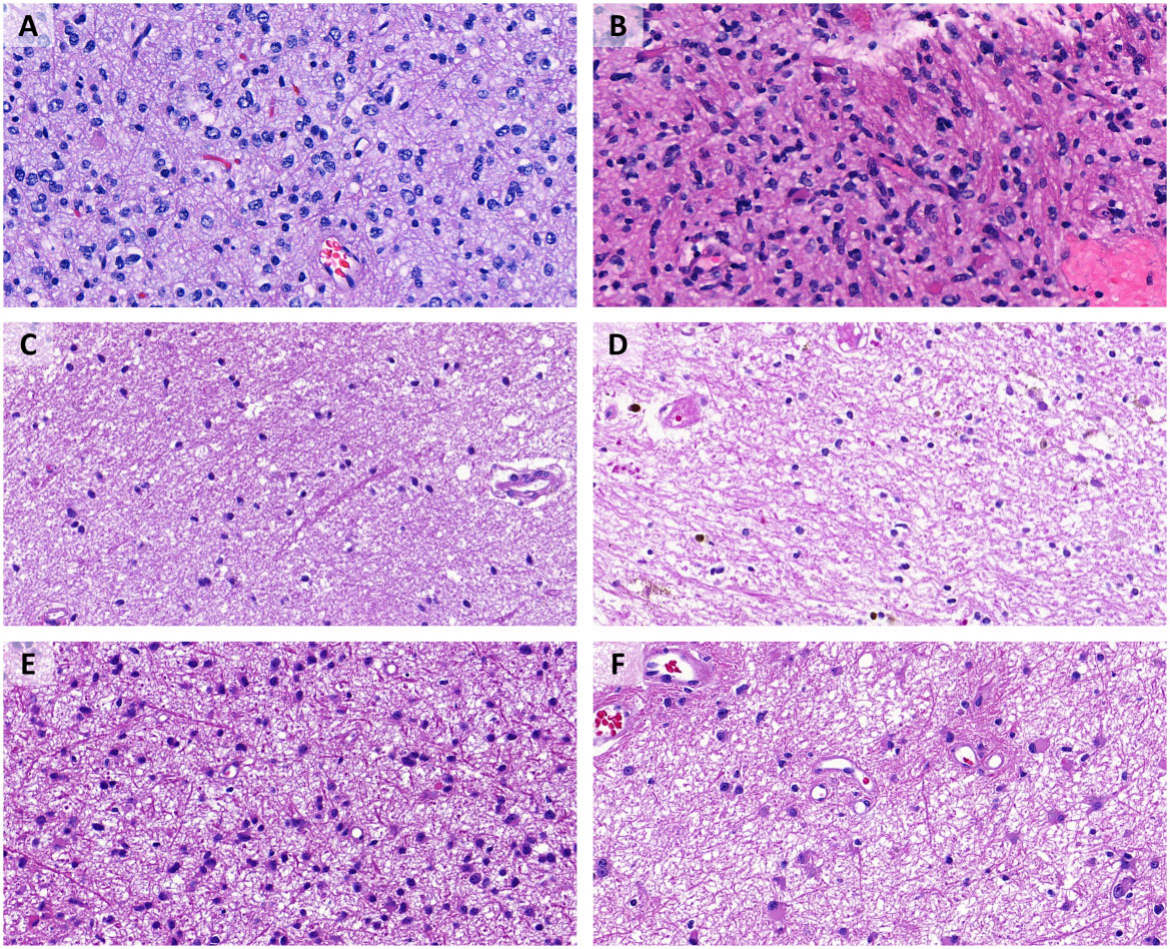
Astrocytoma versus astrocytosis experiment. **(A)** IDH-mutant astrocytoma, correctly recognized by both platforms. **(B)** IDH-mutant astrocytoma, erroneously identified as gliosis by Microsoft Custom Vision, correctly recognized by Google AutoML. **(C)** Gliosis, correctly recognized by both platforms. **(D)** Gliosis, erroneously identified as astrocytoma by Microsoft Custom Vision, correctly recognized by Google AutoML. **(E)** IDH-mutant astrocytoma, correctly recognized by both platforms. **(F)** IDH-mutant astrocytoma, erroneously identified as gliosis by Google AutoML, correctly recognized by Microsoft Custom Vision.

A total of 770 images from 79 cases with an IDH-mutant astrocytoma were assigned to the group called “astrocytoma”; 286 images from 47 cases with gliosis were attributed to the group “gliosis.” Based on the images in these 2 separated groups, the model was trained to recognize and distinguish images from IDH-mutant gliomas and gliosis. Considering its own statistics, Google AutoML was able to identify testing images as astrocytoma or gliosis with a precision and recall rate of 94.1%. For the same task, Microsoft Custom Vision reached - also based on its own statistics - a precision and recall rate of 96.7% (Table 2 and Fig. 3).

**Table 2.** Accuracy of the algorithms as determined by the machine learning platform

Experiment	Precision and recall by Google Auto ML	Precision and recall by Microsoft Custom Vision
Astrocytoma vs astrocytosis	94.1%	96.7%
Glioma vs brain metastasis	98.4%	96.9%
Prediction of 1p/19q-status in IDH-mutant tumors	88.6%	83.5%
Prediction of 1p/19q-status in IDH-mutant tumors without “Oligoastrocytoma”	90.9%	88.5%
Negative control experiment: even vs odd accession numbers	80.8%	78%
Negative control experiment: astrocytoma vs astrocytoma	45.3%	45.5%

### Glioma versus brain metastasis

The group “glioma” contained a total of 1216 images from 121 cases with an IDH-mutant glioma as follows: 720 images from 74 cases of IDH-mutant astrocytoma, CNS WHO grades 2–3, 50 images from 5 cases of IDH-mutant astrocytoma, CNS WHO grade 4, and 446 images from 42 cases of oligodendroglioma. The group “brain metastasis” consisted of 577 images from 57 cases with a brain metastasis as follows: 162 images from 14 cases of small cell lung cancer brain metastasis, 208 images from 21 cases of breast carcinoma brain metastasis, and 207 images from 22 cases of melanoma brain metastasis. The distinction of the 2 entities was performed with a precision and recall rate of 98.4% on Google AutoML and 96.9% on Microsoft Custom Vision (Table 2).

To verify whether the predictions and recall rate stated by Google AutoML and Microsoft Custom Vision were adequate, we performed our own statistics. We trained the model again with a slightly reduced selection of the images mentioned above and kept the rest as testing images with the aim of testing test the model with images from cases that are not included in the training set (Table 3). For this, we used 1028 images from 103 cases with an IDH-mutant glioma (620 images from 64 cases of IDH-mutant astrocytoma, CNS WHO grades 2–3, 40 images from 4 cases of IDH-mutant astrocytoma, CNS WHO grade 4, and 368 images from 35 cases of oligodendroglioma). There were 477 images from 47 cases with a brain metastasis as follows: 132 images from 11 cases of small cell lung cancer brain metastasis, 178 images from 18 cases of mammary carcinoma brain metastasis, and 167 images from 18 cases of melanoma brain metastasis.

**Table 3.** External validation of the “Glioma vs metastasis” classifiers in 2 runs

	Auto ML (1st run)		Auto ML (2nd run)		Microsoft Custom Vision	
	Results	Mean “certainty” of prediction	Results	Mean “certainty” of prediction	Results	Mean “certainty” of prediction
Correctly recognized as glioma	179/188 = 95%	98.9%	181/188 = 96%	98.6%	185/188 = 98%	98.7%
Erroneously recognized as brain metastasis	9/188 = 5%	79.0%	7/188 = 4%	90.6%	3/188 = 2%	78.5%
Correctly recognized as a brain metastasis	95/100 = 95%	96.9%	98/100 = 98%	97.6%	97/100 = 97%	97.0%
Erroneously recognized as glioma	5/100 = 5%	80.9%	2/100 = 2%	91.6%	3/100 = 3%	81.0%

Google AutoML provided slightly different results (despite identical images submitted); results with Microsoft Custom Vision were identical between the 2 runs.

After having trained the model with those images, we subsequently tested it with 188 images from 18 cases with an IDH-mutant glioma (100 images from 10 cases of IDH-mutant astrocytoma, 10 images from 1 case of an IDH-mutant astrocytoma, CNS WHO grade 4, and 78 images from 7 cases of oligodendroglioma) and with 100 images from 10 cases with a brain metastasis (30 images from 3 cases of small cell lung cancer brain metastasis, 30 images from 3 cases of mammary carcinoma brain metastasis, and 40 images from 4 cases of melanoma brain metastasis).

In the first trial, Google AutoML recognized 179 out of 188 images correctly as glioma, which corresponds to a recall rate of 95%. In the second trial, it recognized 181 out of 188 images, which corresponds to a recall rate of 96%. The average precision was 98.9% in the first trial and 98.6% in the second trial. For brain metastases, with a precision of 96.9% in the first trial and 97.6% in the second trial, Google AutoML correctly recognized 95 and 98 of 100 images in the first and second trial, which corresponds to a recall rate of 95% and 98%, respectively. Microsoft Custom Vision achieved the same results in both trials, that is, a recall rate of 98% in the recognition of glioma (185 out of 188), with a precision of 98.7%; for brain metastases, the recall rate was 97% (97 out of 100), with a precision of 97%. These results largely confirm results based on the statistics made by Google AutoML and Microsoft Custom Vision.

**Figure 4.**
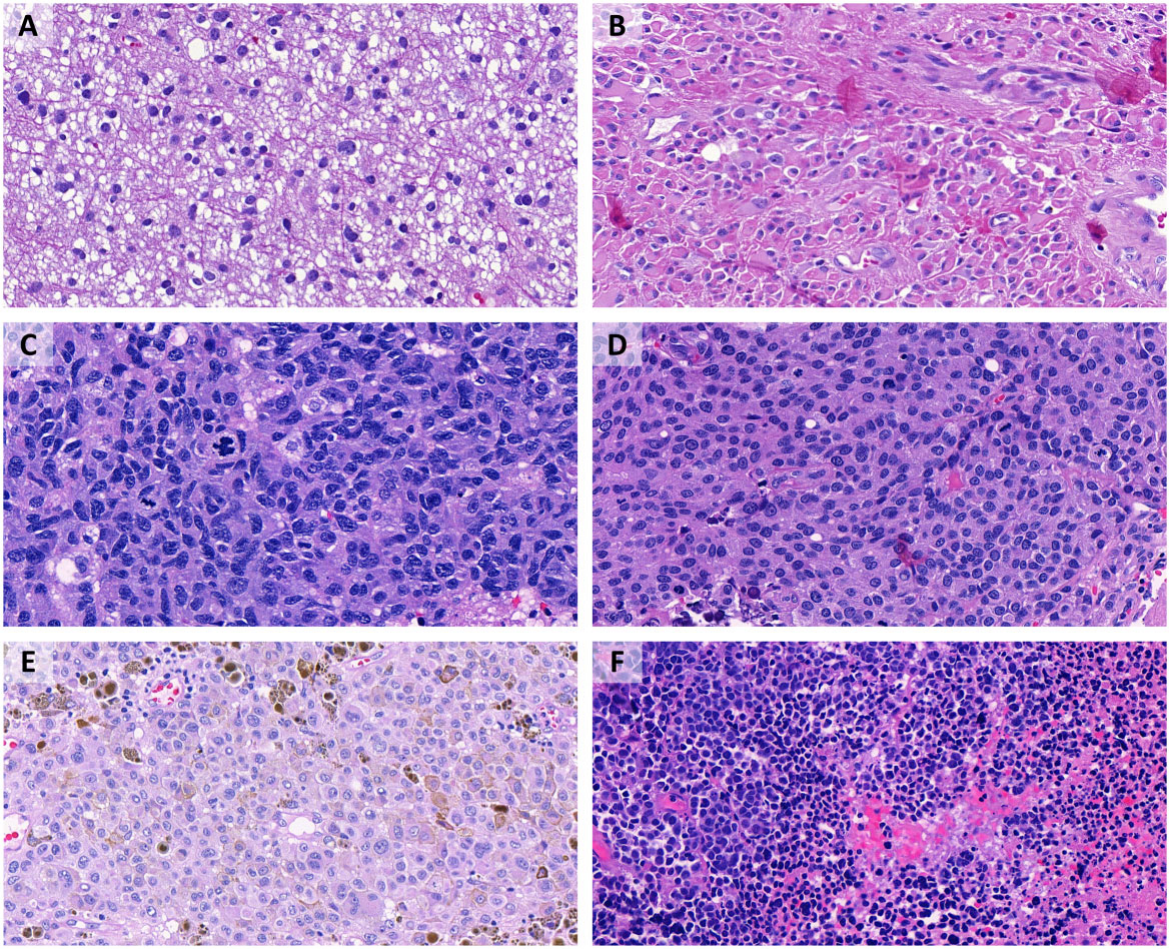
Glioma versus brain metastasis experiment. **(A)** IDH-mutant astrocytoma, correctly recognized as glioma by both platforms. **(B)** Oligodendroglioma, misclassified as brain metastasis by Google AutoML, possibly due to the epithelioid morphology with abundant eosinophilic cytoplasm. **(C)** Brain metastasis (small cell lung carcinoma), correctly recognized as brain metastasis by both platforms. **(D)** Brain metastasis (breast carcinoma), erroneously identified as glioma by Google AutoML. **(E)** Brain metastasis (melanoma), erroneously identified as glioma by Microsoft Custom Vision. **(F)** Brain metastasis (melanoma), erroneously identified as glioma by both platforms. This was the only image of our separate testing set that was misclassified by both algorithms.

Despite the high recall rates and precision, the alleged “certainty” of the few misclassified images was also high. On Google AutoML, the mean certainty of erroneously identified brain metastasis was at 79% in the first trial and 90.6% in the second trial and for erroneously identified glioma at 80.9% in the first trial and at 91.6% in the second trial. The mean precision of the misclassified images on Microsoft Custom Vision accounted for 78.5% of erroneously identified brain metastasis and 81% of erroneously identified glioma in both trials. Interestingly, only one of the images was misclassified by both programs, AutoML and Microsoft Custom Vision (Fig. 4F).

To investigate the numbers of training images required for optimal classification, we compared different models with 50 or 100 images per class. Additionally, we varied the number of cases from whom the images had originated. The performance of Microsoft Custom Vision and of Google AutoML, based on their own statistics, was considerable even if the different classes only contained 50 images. Nevertheless, models with 100 images per group always showed better or at least equal precision and recall rates compared to models with only 50 images. In this context, it must be said that when the training was repeated several times, some variations in the results were observed, particularly if the models only contained 50 images per category. Furthermore, a larger variety of cases did not improve precision and recall rate. It seems that this is due to the fact that the training images and the testing images (on which the statistics made by Google AutoML and Microsoft Custom Vision were based) originated from the same cases. Nonetheless, it can be assumed that for testing images from unknown cases, a higher number and variety of training images and cases should lead to higher precision and recall rates.

### Prediction of 1p/19q co-deletion in IDH-mutant gliomas

**Figure 5.**
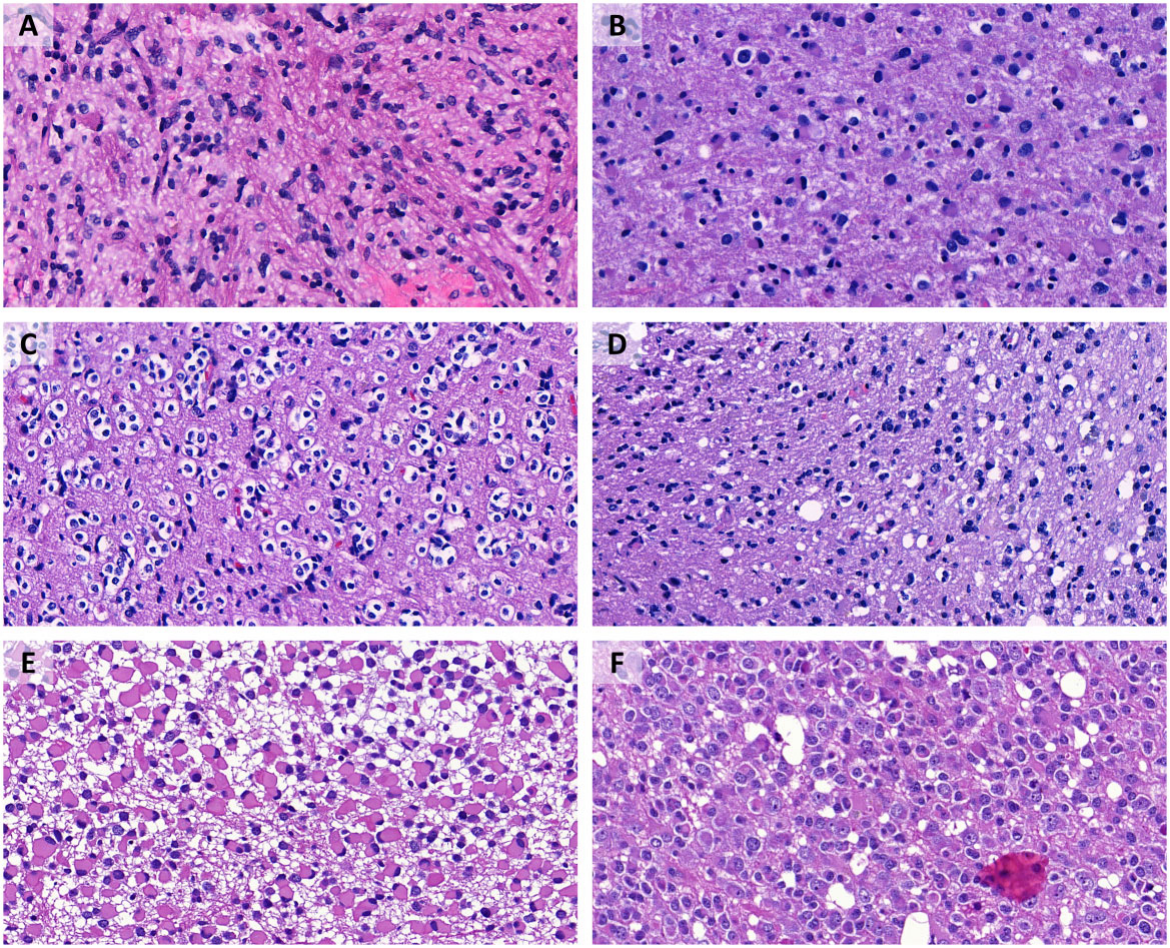
Prediction of 1p/19q co-deletion in IDH-mutant gliomas. **(A)** IDH-mutant astrocytoma, correctly predicted not to be co-deleted by both platforms. **(B)** IDH-mutant astrocytoma, CNS WHO grade 4, erroneously predicted to be co-deleted by Google AutoML, correctly recognized by Microsoft Custom Vision. **(C)** Oligodendroglioma, correctly recognized by both platforms. **(D)** Oligodendroglioma, erroneously predicted not to be co-deleted by both platforms. **(E)** IDH-mutant astrocytoma, correctly recognized by both platforms. **(F)** Oligodendroglioma, erroneously predicted not to be co-deleted by Microsoft Custom Vision, correctly recognized by Google AutoML.

A total of 770 images from 79 cases of IDH-mutant astrocytoma (including 50 images from 5 cases of IDH-mutant astrocytoma, CNS WHO grade 4), comprised the group “astrocytoma”; 446 images from 42 cases of oligodendroglioma with 1p/19q co-deletion comprised the group “oligodendroglioma.” Twenty-six of these total 121 cases initially were given the histological diagnosis of an oligoastrocytoma. Of these, sixteen cases subsequently were diagnosed as IDH-mutant astrocytoma and 10 were diagnosed as oligodendroglioma. Both programs had some difficulties distinguishing an image as either astrocytoma or oligodendroglioma: Google AutoML showed a recall rate and precision of 88.6%, Microsoft Custom Vision of 83.5%, both based on their own statistics (Fig. 5).

To verify whether these results were due to histologically more equivocal “oligoastrocytomas,” the experiment was repeated without the 257 images from the 26 cases with the initial diagnosis of an oligoastrocytoma. Consequently, the group “astrocytoma” was composed of 613 images from 63 cases of IDH-mutant astrocytoma (CNS WHO grades 2–4) and 346 images from 32 cases of “oligodendroglioma.” The exclusion of the images from cases with the original diagnosis of “oligoastrocytoma” led to slightly better results as follows: Precision and recall rate from Google AutoML were at 90.9% and from Microsoft Custom Vision at 88.5%, again based on their own statistics.

### Multiple classes

Because a pilot experiment to test whether the ML platforms’ performance would be approximately similar for experiments with multiple classes or multiple labels, we included all images from the above experiments and assigned them applicable labels of “astrocytoma,” “oligodendroglioma,” “glioma,” “metastatic small cell carcinoma,” “metastatic breast carcinoma,” “metastatic melanoma,” “brain metastasis,” or “gliosis.” We also included 200 images from 38 other cases with unrelated pathologies and labeled them “other.”

For this task, Google Auto ML reached a precision of 88.3% and a recall rate of 85% (based on its own statistics). By far the most misclassifications were due to images of gliosis that were erroneously misclassified as glioma. On the same task, Microsoft Custom Vision reached a precision of 79.5% and a recall rate of 90.1% (based on its own statistics). Without the group “other,” Microsoft Custom Vision reached a slightly increased precision of 81.1% and a recall rate of 90.1%.

With Microsoft Custom Vision we repeated the same experiment with a smaller number of groups, that is, “astrocytoma,” “oligodendroglioma,” “glioma,” and “brain metastasis.” Each group consisted of the above-mentioned number of images and cases. In this task, Microsoft Custom Vision reached a better precision of 87.1, but an inferior recall rate of 86.3%.

### Negative control experiments

As a negative control experiment, we defined 2 classes composed of 251 images of IDH-mutant astrocytoma from 26 cases with even pathology accession numbers and 249 images of IDH-mutant astrocytoma from 25 cases with odd pathology accession numbers. Contrary to the expectations of a control task, Microsoft Custom Vision reached precision and recall rates of 78% and Google AutoML precision and recall rates of 80.8%. To verify those results, we retested the models doing our own statistics with separate testing images from cases that were not part of the training images. Google AutoML recognized the images with a “mean certainty” of 76.3%, 82% were recognized “correctly” as odd, only 34% were recognized “correctly” as even. Microsoft Custom vision reached a “mean certainty” of 72%, 88% were recognized “correctly” as odd, only 6% were recognized as even. These results show that it seems possible that the automated ML programs may be able to find similarities between images that humans are not able to see and that may direct diagnostic testing on the wrong pathway.

In a second control task, each control group contained images from the same cases (335 images of IDH-mutant astrocytoma from 67 cases in one group and 335 different images of IDH-mutant astrocytoma from the same 67 cases in the other group). Here, Google AutoML showed a precision and recall rate of 45.3% and Microsoft Custom Visio a rate of 45.5%.

As an additional negative control experiment, we trained the 2 platforms with 2 training sets, each consisting of the exact same 100 images from 10 cases, but rotated by 180° in one of the groups. In this control task, Microsoft Custom Vision reached a precision and recall rate of 57.5%. On Google AutoML, the experiment could not be performed, because the control group images were identified as rotated versions of the original images.

## DISCUSSION

In this study, we found that 2 platforms for code-free ML, Google AutoML and Microsoft Custom Vision, classified histological images with an accuracy potentially useful for diagnostic purposes. Both user interfaces are user-friendly and accessible to investigators without coding experience while offering adequate versatility to address image classification tasks of clinical interest. Such an approach to code-free ML may have a significant potential to bypass the obstacle of requiring qualified staff with both training in data science and sufficient understanding of histopathology to implement code-based solutions. Importantly, this study is one of the first to assess the performance of more than one platform for code-free learning on the same datasets of histopathological images. In part, the potential utility of this approach relates to the low threshold for beginners in the field. On the other hand, the existence of application programming interfaces would facilitate real-life implementation of such classifiers.

We found independent validation of the accuracies predicted by each platform to be important for several reasons. Both Google AutoML and Microsoft Custom Vision tended to overestimate accuracy when they were trained with inadequately low numbers of images. More critically, when exposed to a nonsense classification without plausible morphological underpinning (i.e. odd vs even accession number), both platforms reported a relatively high alleged accuracy (78% and 80%, respectively), which was presumably due to overfitting for random differences between the respective cases. Indeed, our independent validation confirmed that the actual accuracy of the classifiers corresponded to pure chance in these negative control experiments.

In contrast, estimated accuracy was close to our assessment of accuracy with external validation datasets, when the platforms had been trained with sufficient numbers of images (i.e. in the order of magnitude of several hundred images per class), and in meaningful experiments. These ranged between 98.4% (Google AutoML in the “Glioma vs Metastasis” task) and 83.5% (Microsoft Custom Vision in the “1p/19q” task). Interestingly, accuracy obtained with the ML platforms paralleled the ease with which humans would presumably perform the different tasks: Accuracy was highest for the distinction between glioma and metastasis, followed by the distinction between gliosis and glioma, while it was lowest for the prediction of 1p/19q status in IDH-mutant gliomas (which can be notoriously difficult, even for experienced neuropathologists).

Interestingly, accuracy for the “1p/19q” task increased when a cohort of cases originally classified as oligoastrocytomas (i.e. morphologically indeterminate for astrocytoma vs oligodendroglioma and thereby for anticipated 1p/19q status) were excluded. The latter finding may suggest that cases that are perceived as ambiguous by neuropathologists, indeed are also morphologically less distinctive for ML. Along the same lines, our approach may be expected to perform less well in classification tasks where there are significant morphological gray zones between tumor types, such as in the case of low-grade glioneuronal tumors. On the other hand, the morphological distinction between astrocytomas and oligodendrogliomas, which both platforms achieved reasonably well, already represents a significant challenge (16). This is evident by the fact that more than a decade of research and extensive molecular characterization were required to convince the neuropathological community that essentially all IDH-mutant gliomas represent either astrocytomas or oligodendrogliomas, with oligoastrocytomas not actually corresponding to a separate tumor type (17, 18).

The strengths of the study design include the existence of a robust ground truth for all 3 experiments and adequate size and heterogeneity of the training sets. Furthermore, external validation of the classifiers and negative control experiments proved to be critical. In particular, the latter type of control is often not reported in studies published to date. Furthermore, we submitted identical datasets to 2 different platforms, both of which tended to perform similarly on each dataset, albeit with a tendency for Google AutoML to predict slightly higher accuracy. As we sought to use standard settings of the 2 user interfaces wherever possible, both interfaces would balance precision and recall within each experiment. Further optimization, such as prioritization of either a greater precision or recall, would be possible depending on the intended use of a classifier but exploring the utility of such an approach was beyond the scope of the present study.

For reasons of feasibility we restricted this study to H&E stains performed in a single laboratory (although over a period of 2 decades). The slides were scanned with one specific device and screenshots were performed at one particular magnification. These restrictions would likely limit the transfer of these image classifiers to other laboratories, which, however, was beyond the scope of the present study.

Another limitation of this study consists of its restriction to classification of entire image files rather than segmentation or object identification, which would prevent the algorithms from being applied directly to whole slide scans. Image classification as a study subject, however, offered several important advantages, in that: (1) it was available in both platforms from the beginning of our study (while object identification was only gradually implemented), (2) it facilitated definition of a robust ground truth for each image, and (3) it allowed for a simple potential workflow (either by manually submitting image files to the classifiers or possibly through application programming interfaces). Furthermore, image classification may be considered easier to implement from a regulatory perspective. An immediately available image classifier might, for example, be used to support intraoperative interpretation or guide the initial choice and/or sequence of ancillary testing without having a direct impact on the definitive diagnosis.

Google AutoML has been used for the classification of histopathological images in a small number of published studies, generally with findings consistent with our observations. A study entitled “A ML model for detecting invasive ductal carcinoma with Google Cloud AutoML Vision,” showed “a score of 91.6% average accuracy” by testing their trained Google AutoML model for the identification of invasive ductal carcinoma on whole slide images. Accuracy was slightly inferior (84.6%) with a validation dataset (19). The authors suggested that a balanced sample size between training groups was an important factor for accuracy, which motivated them to perform “data augmentation” by rotation of images even though the actual benefit of such an artificial inflation of training groups remains unclear. With regard to neuropathological applications, Google AutoML used for distinction between tufted astrocytes, astrocytic plaques and neuritic plaques on phospho-tau immunohistochemical-stained images from the motor cortex achieved precision and recall rates between 98% and 100% (20). Google AutoML was trained on microphotographs from testicular biopsies to class them into 1 of 4 Johnsen score groups (a histological score for assessment of spermatogenesis). The authors found out that expanded images focusing on “characteristic areas with seminiferous tubules” resulted in a much higher precision and recall (99.5% average precision, 96.23% recall) than nonexpanded images on 400× magnification (82.6% average precision, 60.96%) (21).

A study published as a preprint compared the performance of Google AutoML to that of a code-based solution (Apple Create ML) with regard in the ability to classify histopathologic images in a series of experiments including normal versus corresponding tumoral tissues, different tumor types, and molecular alteration (presence of KRAS mutation) (22). In that study, recall and precision were between approximately 80% and 100% without significant differences between the 2 platforms. Puri et al used Google AutoML for classification of histological patterns of drug-induced liver injury according to the causative drug and obtained 92.9% accuracy, though without an external validation cohort (23). Of note, a concept of a locally run code-free pipeline for deep learning based on open-source software has also been described (7). In their proof-of-concept study, the authors used the pipeline for image segmentation tasks but it would be expected to be equally able to perform other tasks such as image classification.

With the exception of the study concerning classification of tau-related lesions mentioned above, neuropathological applications of ML-based image classification for the most part have used code-based solutions. Fields of application have included glioma classification based on H&E-stained slides (24), classification of tauopathies (25), cerebral amyloid pathologies (26), white matter pathology in tauopathies (27) or microglia detection and morphology (28).

A number of studies have assessed the performance code-free platforms for classification of nonhistological medical images. Faes et al used publicly available open source datasets including retinal fundus images, optical coherence tomography (OCT) images; images of skin lesions, and chest X-ray to assess the performance of Google AutoML (29). They found accuracy to be better for binary classifications than for tasks with multiple classes. Korot et al performed a comparative study of 6 different platforms (including Google AutoML and Microsoft custom vision) on OCT images and fundus photographs with regard do diabetic retinopathy (8). The major platforms generally showed a similar performance; of note, the authors found published bespoke algorithms not to perform systematically better than code-free algorithms. The same group used Goggle AutoML for prediction of sex from photographs achieving 86.5% accuracy in the training cohort and 78.6% accuracy in the external validation cohort (9). Antaki et al found an interactive application within MATLAB to allow 2 ophthalmologists without previous coding experience to build a ML mode for proliferative vitreoretinopathy (30).

Our findings are in accordance with the published literature in that we found code-free ML to be easily accessible to investigators without previous coding experience while being sufficiently accurate in diagnostically meaningful tasks to be of potential utility in real-life applications. In comparison to the published literature, the present study highlights a number of points that have rarely been addressed: (1) We found external validation of the predicted recall and precision to be crucial, as both platforms tended them when sample sizes were small, while we predicted values to be accurate when the algorithms were trained with a sufficient number of images (in the order of several hundreds). (2) Along the same line, we found negative control experiments to be important, as both platforms predicted an accuracy of around 80% when trained with nonsense input (even vs odd accession number); also, the external validation cohort showed the true accuracy to be much lower. (3) We systematically compared the performance of 2 major code-free ML platforms and found minor differences in the user interface and the workflow but an overall comparable performance throughout a series of experiments.

In summary, we conclude that code-free ML platforms enable researchers without previous coding experience to perform meaningful experiments and develop classifiers with sufficient accuracy for potential real-life applications at low costs. Application programming interfaces in principle allow for integration of these classifiers in external work-flows. These classifiers might most easily be used in settings that pose no or minor regulatory issues, for example, in research settings, for automated annotation of image archives or possibly in diagnostic work-flows with some redundancy; for example, the decision in which order a certain series of tests should be requested.

Platforms for code-free ML may arguably be of even more interest to neuropathologists as a training tool (and playground) as they significantly lower the threshold in order to make their first steps in the field. Their morphological competences as well as their understanding of the biology and clinical consequences of lesions of the nervous system will be a key factor for successful implementation of ML-based image classification in general. Therefore, hands-on experience that may be obtained more easily through code-free platforms will likely be of utmost importance.

## Supplementary Material

nlac131_Supplementary_Data
