# Supplementary material for: Code-free machine learning for classification of central nervous system histopathology images
Source: J Neuropathol Exp Neurol. 2023 Feb 3;82(3):221–30. doi: 10.1093/jnen/nlac131 (PMC9941804; doi:10.1093/jnen/nlac131)
Supplement: nlac131_Supplementary_Data [file nlac131_supplementary_data.pdf]

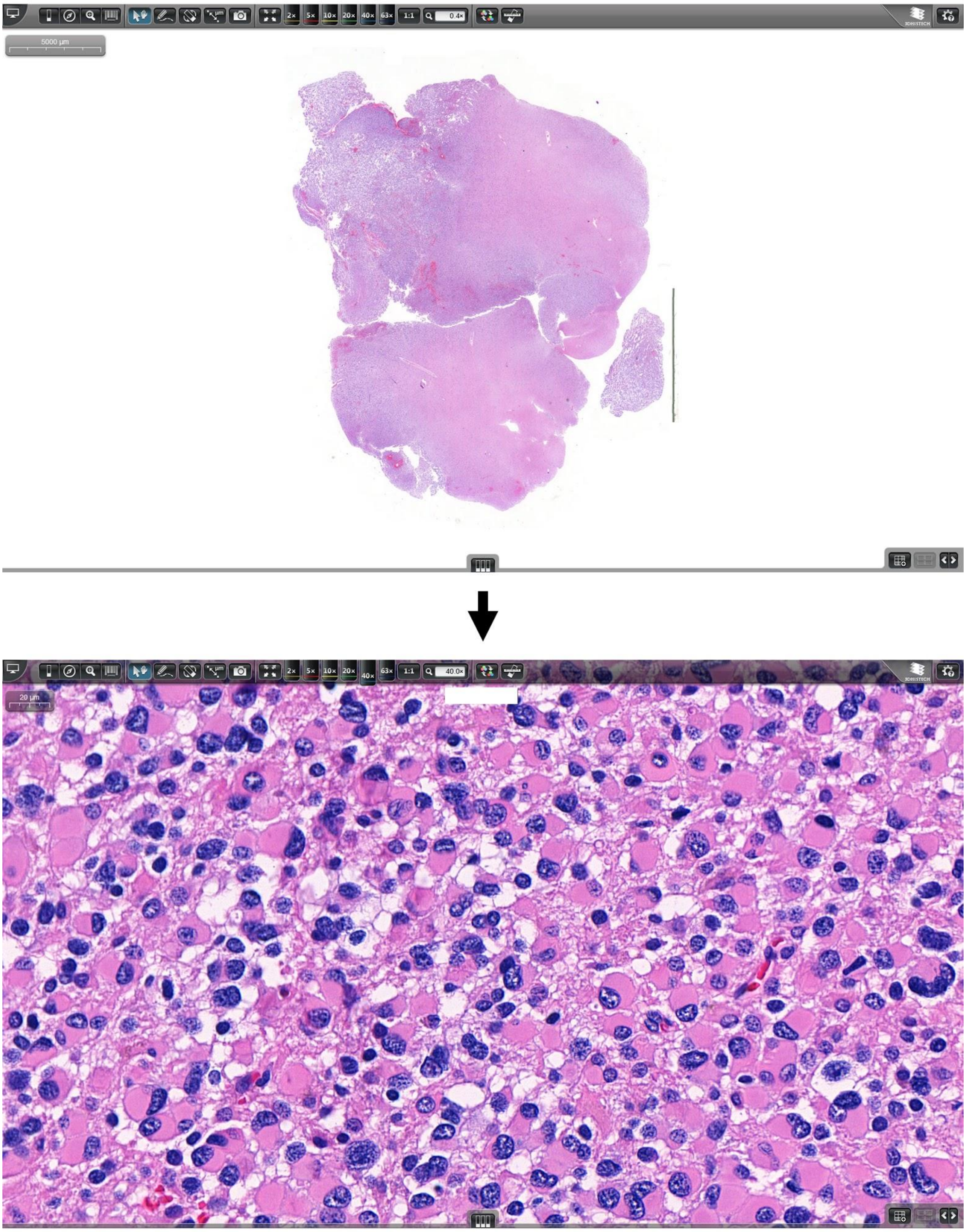
5000 µm
0.4×
20 µm
40.0×

Supplementary figure (generation of histopathological images). Scans (MRXS file format) were reviewed with the CaseViewer (3DHistech, Budapest, Hungary) software. Snapshots of representative pathological regions of interest (ROI) of the scan were made at a nominal 400x magnification (40x “objective”) in JPEG file format.
